# Effect of sedated colonoscopy with different cost coverage on improving compliance with colorectal cancer screening in China

**DOI:** 10.3389/fonc.2023.1156237

**Published:** 2023-07-04

**Authors:** Lin Zhuo, Yunxin Kong, Siting Chen, Yue Ma, Ting Cai, Jianqiang Pan, Xiuying Wang, Yihuan Gao, Hang Lu, Xinyue Li, Hongying Zhao, Louisa Mackay, Wendi Dong, Lang Zhuo, Dong Dong

**Affiliations:** ^1^School of Public Health, Xuzhou Medical University, Xuzhou, China; ^2^Department of Endocrinology, Peking University People′s Hospital, Beijing, China; ^3^Cancer Prevention Office, Xuzhou Cancer Hospital, Xuzhou, China; ^4^School of Management, Xuzhou Medical University, Xuzhou, China; ^5^Department of Nephrology, Xuzhou Central Hospital, Xuzhou, China; ^6^Department of Nephrology, Xuzhou Clinical College of Xuzhou Medical University, Xuzhou, China; ^7^Department of Medical Oncology, Xuzhou Cancer Hospital, Xuzhou, China; ^8^School of Clinical Medicine, Jiangsu University, Zhenjiang, China

**Keywords:** colorectal cancer, screening, sedated colonoscopy, compliance, cost coverage

## Abstract

**Background:**

Colorectal cancer is the third most common cancer worldwide. Colonoscopy is the gold standard for colorectal cancer screening. However, the colonoscopy participation rate in China is much lower than that in Europe and the United States. As only non-sedated colonoscopies are offered in colorectal cancer screening programs in China, the absence of sedation may contribute to this gap.

**Methods:**

To explore the effect of free and partially participant-paid sedated colonoscopy on improving colorectal screening participation, we conducted a cross-sectional study under the framework of the Cancer Screening Program in Urban China in Xuzhou from May 2017 to December 2020. The Quanshan district was set as the control group and provided free non-sedated colonoscopy, the Yunlong district was set as a partial cost coverage group and offered partially participant-paid sedated colonoscopy, and the Gulou district was set as the full cost coverage group and offered free sedation colonoscopies. Multivariate logistic regression was used for multivariate analysis of colonoscopy participation and colorectal lesion detection rates between the groups.

**Results:**

From May 2017 to May 2020, 81,358 participants were recruited and completed questionnaire, 7,868 subjects who met high-risk conditions for CRC were invited to undergo colonoscopy. The colonoscopy participation rates in the control group, partially cost coverage, and full cost coverage groups were 17.33% (594/3,428), 25.66% (542/2,112), and 34.41% (801/2,328), respectively. Subjects in the partial and full cost coverage groups had 1.66-fold (95% CI: 1.48–1.86) and 2.49-fold (95% CI: 2.23–2.76) increased rates compared with those in the control group. The adjusted PARs for the partially and the full cost coverage group was 9.08 (95% CI: 6.88–11.28) and 18.97 (95% CI: 16.51–21.42), respectively. The detection rates of CAN in the control, partial-cost coverage, and full-cost coverage groups were 3.54% (21/594), 2.95% (16/542), and 5.12% (41/801), respectively. There were no significant differences in the detection rates between the group. However, sedated colonoscopy increases costs.

**Conclusion:**

Sedated colonoscopy increased colonoscopy participation rates in both the partial and full cost-covered groups. A partial cost coverage strategy may be a good way to increase colorectal cancer participation rates and quickly establish a colorectal cancer screening strategy in underfunded areas.

## Introduction

Colorectal cancer (CRC) is the third most common cancer and second leading cause of cancer-related deaths worldwide, with an estimated 1.9 million more new cases and 935,000 deaths in 2020 (1). In China, CRC is the fifth most common cancer in both men and women and is a major public health issue (2). Most CRC occur through the “adenoma-carcinoma” pathway, which usually lasts 5–10 years (3, 4). Screening and early intervention have been shown to be effective in improving survival and preventing CRC development (5, 6).

To reduce cancer incidence and mortality, many countries and regions, including the United States and Europe, have established national colorectal cancer screening programs. The Chinese government also initiated the population-based Cancer Screening Program in Urban China (CanSPUC) in October 2012, which targeted common cancers that are most prevalent in urban areas, including CRC. Eligible participants were recruited from communities in the study regions and invited to undergo cancer screening free of charge. Participants were first invited to take a cancer risk assessment by an established Clinical Cancer Risk Score System, and those who were evaluated to be at high risk for CRC were recommended to undergo subsequent colonoscopy at tertiary-level hospitals designated by the program. The CanSPUC recruited 1,381,561 eligible participants aged 40–69 years from 16 provinces in China from 2012 to 2015, and 182,927 participants were evaluated to be at high risk for CRC; however, only 25,593 participants underwent colonoscopy as recommended, with a participation rate of 14.0% (7). This colonoscopy participation rate is much lower than the 22.9% (Netherlands) to 60.7% (Norway) reported in the Nordic-European Initiative on Colorectal Cancer (NordICC) study conducted in four European countries (Norway, the Netherlands, Poland, and Sweden) (8) and 60.8% among adults aged 50–75 years in the United States (9), seriously affecting the effectiveness of colorectal cancer screening in China.

As only non-sedated colonoscopies are offered in the CanSPUC, whereas sedated colonoscopies are commonly offered in Europe and the United States (8, 10–12), the absence of sedation may contribute to the gap in colonoscopy participation rates between China, Europe, and the United States. Sedated colonoscopy has many advantages, such as analgesia and anxiolysis (10–12), which may be important for improving colonoscopy participation, as pain and anxiety are partly responsible for poor colonoscopy participation. However, sedated colonoscopy also increases the risk of hypotension and hypoxemia and requires a specially qualified medical team comprising nurses, anesthetists, and incurs additional costs compared with non-sedation colonoscopy (10–12). Colorectal cancer screening in CanSPUC is currently paid for by a special government fund but will be covered by the Basic Medical Insurance Pooling Fund in the future. China’s Basic Medical Insurance system is divided into pooling and individual accounts. The pooling fund account is funded by employers and national financial subsidies and is shared by all insured persons, while individual accounts are funded by individuals and owned by themselves. In recent years, the participation rate in China’s Basic Medical Insurance has remained stable at approximately 95%. For mass screening programs, it is not possible to increase the cost of providing free-sedated colonoscopy when its effectiveness is uncertain. This problem may have been solved by the participants paying for additional sedation at their own discretion. However, the results of a study in Guangzhou, China, showed that the participation rate in free colonoscopy was higher than that in paid colonoscopy (20.27% *vs*. 10.70%), and most participants could not accept paying more than 300 yuan for CRC screening (13, 14).

To explore the effect of free and partially participant-paid sedated colonoscopy on improving colorectal screening participation and advise on health policy improvements, we conducted a cross-sectional study under the framework of the CanSPUC in Xuzhou. Xuzhou is the central city of the Huaihai Economic Zone (which has a population of 119 million, covers an area of 178,000 km^2^, and consists of 20 cities), located at the junction of four provinces (Jiangsu, Anhui, Shandong, and Henan), southeast of the North China Plain, and a gateway to East China. The participation rate of Basic Medical Insurance in Xuzhou was approximately 98.5%. From May 2017 to December 2020, Colorectal cancer screening was conducted in the Quanshan, Yunlong, and Gulou districts, and different cost coverage strategies were provided in each district.

## Methods

### Study design and population

We conducted this study using the CanSPUC framework. CanSPUC is an ongoing national cancer screening program in the urban areas of China, and Xuzhou joined this program in August 2014. Briefly, a cluster sampling method was adopted to conduct simple random sampling of the community as a group in the main urban area of Xuzhou. Residents living in selected communities aged 40–74 years were approached by trained staff *via* phone calls and personal encounters. After obtaining signed written informed consent, all eligible participants (aged 40–74 years, local permanent resident population, no major diseases) were interviewed by trained staff to collect information about their exposure to risk factors and to evaluate their cancer risk using conditions set by the National Cancer Center. To optimize the use of limited colonoscopy resources and to enhance the detection rate of colorectal neoplasia, only participants who met the high-risk conditions for CRC were recommended to undergo colonoscopy at Xuzhou Cancer Hospital, designated by the programmer free of charge. All data collection processes were conducted using an information system built specifically for CanSPUC by the National Cancer Center.

From May 2017 to May 2020, colorectal cancer screening was conducted in the Quanshan, Yunlong, and Gulou districts. Different colonoscopy and cost coverage strategies were provided for each district. The Quanshan District was set as the control group and provided free non-sedated colonoscopy according to the CanSPUC technical protocol. The Yunlong district was set as a partial cost coverage group and offered partially participant-paid sedated colonoscopy, CanSPUC funding paid for colonoscopy, and participants paid for their own sedation (about 376 yuan, can pay with Basic Medical Insurance Personal Account). Participants who refused to pay for sedation also had the option of undergoing an unsedated colonoscopy free of charge. The Gulou District was set as full cost coverage group and offered free sedation colonoscopies; all costs were covered by CanSPUC funds. Participants who refuse to undergo sedation can undergo free non-sedation colonoscopies.

**Figure 1:**
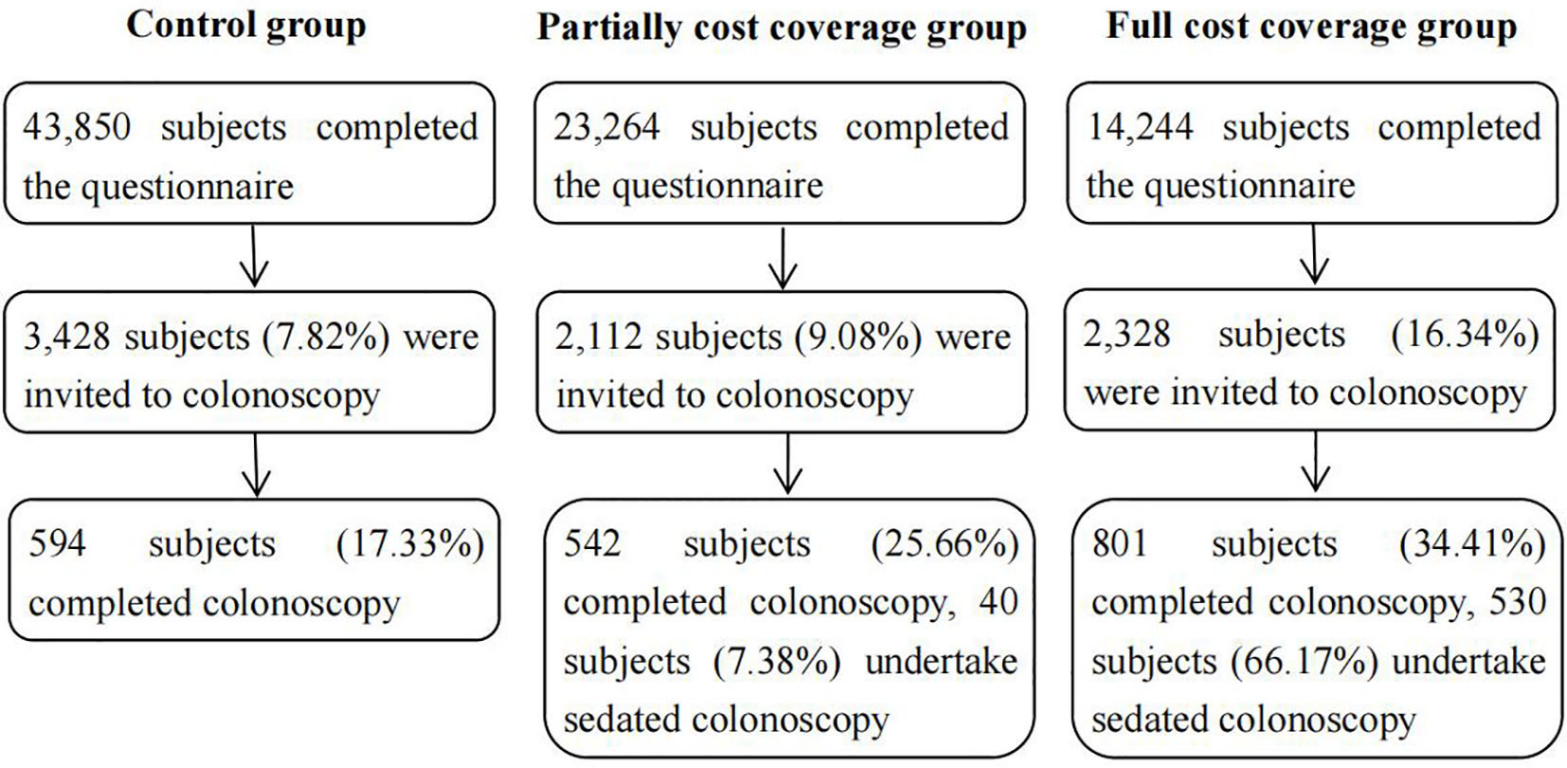
Recruitment flow chart of this study.

A total of 81,358 participants were recruited and completed the questionnaire; 7,868 subjects (9.67%) who met the high-risk conditions for CRC were invited to undergo colonoscopy, and 1,937 subjects (24.62%) completed colonoscopy. A recruitment flowchart is shown in **Figure 1**. This study was approved by the Ethics Committee of Xuzhou Cancer Hospital (approval number: 2018-02-23-H01).

### Sample size

The colonoscopy participation rate and number of subjects invited for colonoscopy in each group were used to calculate the power. When the colonoscopy participation rate of the control group, the partially cost coverage group and the full cost coverage group were 17.33%, 25.66%, and 34.41%, respectively, and the number of subjects invited for colonoscopy were 3,428, 2,112, and 2328, respectively, the power of comparison of colonoscopy participation rate between groups was 0.999. This means that the sample size of this study was sufficient to compare the differences in colonoscopy participation rates between the groups.

### Colonoscopy screening

The nature, benefits, and risks of colonoscopy were explained to all subjects prior to the examination, and a colonoscopy risk notification form was signed. We used polyethylene glycol (HYGECONR, Jiangxi Hygecon Pharmaceutical Co., Ltd., China) as a standard bowel preparation regimen for all participants, and an electrocardiogram was also performed before colonoscopy to prevent unexpected events. Propofol (Yangzijiang; Yangzijiang Pharmaceutical Group Co., Ltd., China) was used as a sedative for subjects selected for sedated colonoscopy. A team of experienced physicians, colorectal surgeons, nurses, and anesthetists performed all colonoscopy procedures at the endoscopy Center of Xuzhou Cancer Hospital. All abnormal findings were pathologically examined in accordance with the clinical procedures, and the results and images were uploaded to the project information system. Colorectal advanced neoplasia (CAN) was the most important abnormal finding and was defined as CRC or any colorectal adenoma measuring 1 cm or more in diameter, high-grade dysplasia, or tubular-villous histologic features. To ensure the quality of the examination, the quality control team, composed of the chief physician and deputy chief physician, reviewed all results.

### Statistical analysis

Statistical analyses were performed using Stata version 16.0. Statistical significance was defines as a two-tailed *P*-value <0.05. The basic characteristics of the study population were first described and compared between the study groups using the Pearson χ^2^ test. The Pearson χ^2^ test was also used for the univariate analysis of colonoscopy participation rates. Multivariate logistic regression was used for multivariate analysis of colonoscopy participation rates and colorectal lesion detection rates between the groups, and adjusted ORs and P-values were reported. Based on the adjusted ORs, the adjusted RRs and PARs were calculated. The cost of colonoscopy in the different groups paid by funds was also calculated.

## Results

### Characteristics of the study population

From May 2017 to May 2020, 81,358 participants were recruited to complete the questionnaire. A total of 7,868 subjects who met the high-risk conditions for CRC were included in the analysis and invited to undergo colonoscopy, including 3,428 in the control group, 2,112 in the partial cost coverage group, and 2,328 in the full cost coverage group. The characteristics of the study population in the different groups are shown in **Table 1**, and all factors were different between the three groups (*P <*0.05).

**Table 1:** Characteristics of the study population in different groups [n (%)].

Factors	Control group (n = 3,428)	Partial cost coverage group (n = 2,112)	Full cost coverage group (n = 2,328)	*P*
Age				<0.001
40–44	287 (8.37)	136 (6.44)	81 (3.48)	
45–49	536 (15.64)	298 (14.11)	198 (8.51)	
50–54	669 (19.52)	363 (17.19)	272 (11.68)	
55–59	696 (20.30)	363 (17.19)	390 (16.75)	
60–64	758 (22.11)	399 (18.89)	333 (14.30)	
65–69	440 (12.84)	358 (16.95)	648 (27.84)	
70–74	42 (1.23)	195 (9.23)	406 (17.44)	
Sex				<0.001
Male	1,567 (45.71)	906 (42.90)	1,533 (65.85)	
Female	1,861 (54.29)	1,206 (57.10)	795 (34.15)	
Education background				0.003
<High school	2,086 (60.85)	1,239 (58.66)	1,442 (61.94)	
High school and equivalent	905 (26.40)	627 (29.69)	572 (24.57)	
≥Postsecondary graduate	437 (12.75)	246 (11.65)	314 (13.49)	
Family history of CRC among the first-degree relatives				<0.001
No	3,005 (87.66)	1,886 (89.30)	2,174 (93.38)	
Yes	423 (12.34)	226 (10.70)	154 (6.62)	
Previously detected colonic polyp				<0.001
No	2,987 (87.14)	1,714 (81.16)	1,783 (76.59)	
Yes	441 (12.86)	398 (18.84)	545 (23.41)	
Fecal occult blood test				<0.001
Negative result or no	3,189 (93.03)	1,983 (93.89)	1,982 (85.14)	
Positive result	239 (6.97)	129 (6.11)	346 (14.86)	

### Colonoscopy participation rate

**Figure 2:**
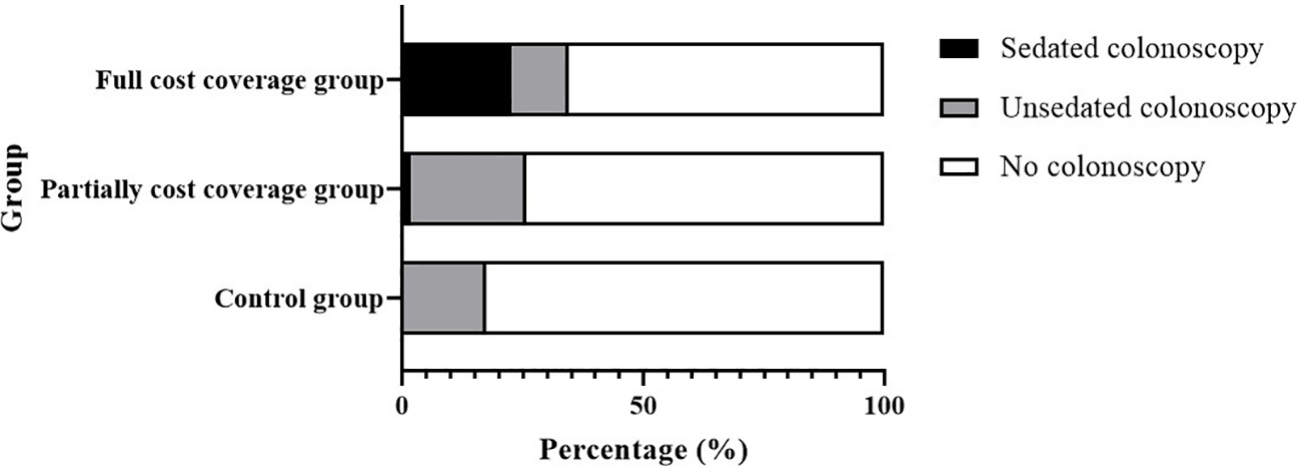
Colonoscopy participation rate and sedated colonoscopy use rate in different groups.

The colonoscopy participation rates in the control, partial cost coverage, and full cost coverage groups were 17.33% (594/3,428), 25.66% (542/2,112), and 34.41% (801/2,328), respectively (**Figure 2**). The sedated colonoscopy uses rates of the partial cost coverage and full cost coverage groups were 7.38% (40/542) and 66.17% (530/801), respectively. In the partial-cost coverage group, all subjects who chose to undergo sedated colonoscopy were paid for their own sedation using the Basic Medical Insurance Individual Account.

### Univariate analysis

In the univariate analysis, group, age, sex, educational background, family history of CRC among first-degree relatives, previously detected colonic polyps, and fecal occult blood test results were all risk factors for colonoscopy participation rate (*P <*0.05), and the results are shown in **Table 2**.

**Table 2:** Univariate analysis of colonoscopy participation rate.

Factors	Participants undertaking colonoscopy (%)	*χ^2^*	*P*
**Group**		219.62	<0.001
Control group	594 (17.33)		
Partially cost coverage group	542 (25.66)		
Full cost coverage group	801 (34.41)		
**Age**		67.98	<0.001
40–44	99 (19.64)		
45–49	283 (27.42)		
50–54	376 (28.83)		
55–59	379 (26.16)		
60–64	390 (26.17)		
65–69	316 (21.85)		
70–74	94 (14.62)		
**Sex**		6.37	0.012
Male	938 (23.41)		
Female	999 (25.87)		
**Education background**		53.44	<0.001
<High school	1,049 (22.01)		
High school and equivalent	569 (27.04)		
≥Postsecondary graduate	319 (32.00)		
**Family history of CRC among** **the first-degree relatives**		49.41	<0.001
No	1,658 (23.47)		
Yes	279 (34.74)		
**Previously detected colonic polyp**		156.97	<0.001
No	1,414 (21.81)		
Yes	523 (37.79)		
**Fecal occult blood test**		184.82	<0.001
Negative result or no	1,612 (22.53)		
Positive result	325 (45.52)		

### Multivariate analysis

In the multivariate analysis, after adjusting for age, sex, educational background, family history of CRC among first-degree relatives, previously detected colonic polyps, and fecal occult blood test results, subjects in the partial cost coverage group and the full cost coverage group had 1.66-fold (95% CI: 1.48–1.86) and 2.49-fold (95% CI: 2.23–2.76) increased rates of colonoscopy participation, respectively, compared with those in the control group (**Table 3**). The adjusted PARs for the partial cost coverage group and full cost coverage group were 9.08 (95% CI: 6.88–11.28) and 18.97 (95% CI: 16.51–21.42), respectively.

**Table 3:** Adjusted ORs, RRs, and PARs of factors associated with participation rate in colonoscopy.

Factors	*OR (95% CI)*	*P*	*RR (95% CI)*	*PAR (%, 95% CI)*
Group				
Control group	*Reference*		*Reference*	*Reference*
Partially cost coverage group	1.78 (1.55–2.04)	<0.001	1.66 (1.48–1.86)	9.08 (6.88–11.28)
Full cost coverage group	2.92 (2.55–3.35)	<0.001	2.49 (2.23–2.76)	18.97 (16.51–21.42)
Age				
40–44	1.95 (1.40–2.70)	<0.001	1.86 (1.38–2.50)	8.51 (4.20–12.78)
45–49	2.89 (2.20–3.80)	<0.001	2.65 (2.08–3.34)	15.17 (11.54–18.76)
50–54	3.05 (2.34–3.98)	<0.001	2.78 (2.20–3.48)	16.21 (12.79–19.59)
55–59	2.75 (2.12–3.57)	<0.001	2.54 (2.01–3.17)	14.31 (11.06–17.52)
60–64	2.97 (2.29–3.85)	<0.001	2.71 (2.16–3.38)	15.70 (12.43–18.93)
65–69	1.98 (1.53–2.56)	<0.001	1.89 (1.49–2.38)	8.76 (5.72–11.84)
70–74	*Reference*		*Reference*	*Reference*
Sex				
Male	*Reference*		*Reference*	*Reference*
Female	1.14 (1.02–1.28)	0.018	1.13 (1.02–1.25)	2.30 (0.39–4.20)
Education background				
<High school	*Reference*		*Reference*	*Reference*
High school and equivalent	1.22 (1.08–1.39)	0.002	1.20 (1.07–1.34)	3.43 (1.26–5.60)
≥Postsecondary graduate	1.53 (1.30–1.80)	<0.001	1.46 (1.27–1.68)	7.58 (4.53–10.62)
Family history of CRC among the first-degree relatives				
No	*Reference*		*Reference*	*Reference*
Yes	1.75 (1.48–2.06)	<0.001	1.64 (1.42–1.88)	10.40 (7.08–13.70)
Previously detected colonic polyp				
No	*Reference*		*Reference*	*Reference*
Yes	1.67 (1.46–1.90)	<0.001	1.57 (1.40–1.76)	9.40 (6.81–11.98)
Fecal occult blood test				
Negative result or no	*Reference*		*Reference*	*Reference*
Positive result	2.22 (1.88–2.62)	<0.001	2.00 (1.74–2.29)	15.54 (11.92–19.12)

OR, Odds ratio; RR, Risk ratio; PAR, Population attributable risk; CI, Confidence interval.

### Detection rate

The detection rates of CAN in the control, partial-cost coverage, and full-cost coverage groups were 3.54% (21/594), 2.95% (16/542), and 5.12% (41/801), respectively. There was no significant difference in the detection rate of CAN between the partial cost coverage group and the control group [*OR* = 0.74 (0.37–1.46), *P* = 0.387], or between the full cost coverage group and the control group [*OR* = 1.21 (0.68–2.12), *P* = 0.515] (**Table 4**).

**Table 4:** Colorectal lesion detection rate in different groups.

Colorectal lesion	Control group	Partial cost coverage group	Full cost coverage group	Partial cost coverage group vs Control group		Full cost coverage group vs Control group	
				*OR * (95% CI)*	*P*	*OR * (95% CI)*	*P*
CAN	21 (3.54%)	16 (2.95%)	41 (5.12%)	0.74 (0.37–1.46)	0.387	1.21 (0.68–2.12)	0.515
CNA	117 (19.70%)	68 (12.55%)	139 (17.35%)	0.58 (0.42–0.80)	0.001	0.80 (0.60–1.06)	0.120
Polyp	76 (12.79%)	88 (16.24%)	130 (16.23%)	1.35 (0.97–1.90)	0.077	1.30 (0.95–1.78)	0.095
Any neoplasm	214 (36.03%)	172 (31.73%)	310 (38.70%)	0.82 (0.63–1.05)	0.113	1.04 (0.83–1.30)	0.738

CAN, Colorectal advanced neoplasm; CNA, Colorectal non-advanced neoplasm; OR, Odds ratio; CI, Confidence interval.

*Adjusted age, sex.

### Cost

The average cost of colonoscopy in the control, partial cost coverage, and full cost coverage groups paid by funds were 266, 266, and 515 yuan, respectively. The cost of colonoscopy needed to detect one case of CAN in each group paid by the fund was 7,524, 9,010, and 10,057 yuan (**Table 5**).

**Table 5:** Cost of colonoscopy in different groups paid by fund (Yuan).

Cost	Control group	Partial cost coverage group	Full cost coverage group
Total cost	158,004	144,172	412,346
Average cost	266	266	515
Cost needed to detect one case of CAN	7,524	9,010	10,057
Cost needed to detect one case of any neoplasm	738	838	1,330

CAN, Colorectal advanced neoplasm.

## Discussion

This is the first study in China to investigate the effect of sedated colonoscopy with different cost coverages on improving compliance with CRC screening in asymptomatic community populations. This study found that sedated colonoscopy increased colonoscopy participation rates in both partial and full cost-covered groups, and there was no statistical difference in the detection rate of CAN compared with the control group. However, sedated colonoscopy also increases costs.

Participation rate is critical for determining the effectiveness of CRC screening. An Australian modeling study (15) showed that increasing colonoscopy participation from 40% to 60% could reduce 37,300 CRC cases and 24,800 CRC deaths over the next 25 years. In a 2012–2015 Chinese study (7), the diagnostic yield was not optimal using colonoscopy screening in high-risk populations, given the relatively low participation rate. But in this study, partial and full cost covered sedated colonoscopy increased participation rates by 9.08% [RR = 1.66 (1.48–1.86)] and 18.97% [RR = 2.49 (2.23–2.76)], respectively, demonstrating the effectiveness of the sedated colonoscopy screening policy in increasing participation rates. However, it is important to note that this study was a real-world field trial conducted in the real world. Due to the limitations of the research conditions, no randomization was conducted, and no balanced comparable control group was available. Community-based randomized controlled trials are recommended to further explore the association between sedated colonoscopy use and colorectal cancer screening participation when conditions permit it.

However, even in the full cost coverage group, the colonoscopy participation rate of the subjects in this study was only 34.41%, which was lower than the 40.0% in Europe (8) and 60.8% in the United States (9). The difference may be related to the basic characteristics of the population, such as the age of the subjects (CanSPUC, 40–74 years, NordICC, 55–64 years, United States, 50–75 years) (7–9). Age is an important factor for colonoscopy participation in countries around the world (7, 8, 16, 17). According to a French analysis (16), uptake was significantly lower in the youngest (50–59 years) and oldest (70–74 years) persons, compared with intermediate ages (60–69 years), with OR = 0.70 (95% CI: 0.63 to 0.77) and OR = 0.82 (95% CI: 0.72 to 0.93), respectively. In a study conducted in Henan, China (17), participants aged 50–64 years were more likely to undergo colonoscopy.

The high rate of colonoscopy participation in the United States is closely related to earlier scientific research and active health policies (18–20). From the mid-1970s to the 1990s, colonoscopy was established as a superior CRC screening modality in the United States (18). In 1997 and 2001, The Balanced Budget Act and Consolidated Appropriations Act were passed to provide access to screening colonoscopies. In 2014, the National Colorectal Cancer Roundtable of the American Cancer Society launched 80% by 2018 (19, 21). Although the target was not met that year, CRC screening rates in the United States have been gradually increasing and achieved good results (9, 20–22). In contrast, China’s nationwide CRC screening program was later launched. Although the National Cancer Center has made many explorations (such as CanSPUC and TARGET-C) (7, 23–25) and wrote the Chinese guidelines for the screening, early detection, and early treatment of colorectal cancer (2020, Beijing) (26), China does not have a national CRC screening policy at present, and the exploration of CRC screening strategies needs to continue to obtain sufficient evidence for CRC screening to be covered by the Basic Medical Insurance Pooling Fund in the future.

In addition, sedated colonoscopy has other advantages, such as easy scope advancement, less examination time, and better cecal intubation rates, which may help further improve the effectiveness of CRC screening (10). However, a study conducted by Liang et al. (27) showed that although sedated colonoscopy improved patient satisfaction, it did not affect the adenoma and polyp detection rates. Sedated colonoscopy did not improve the detection rate of advanced neoplasms and polyps in this study. However, from another perspective, the increase in colorectal cancer screening participation caused by sedated colonoscopy did not dilute the detection rate of colorectal lesions. Studies have also shown that the use of sedated colonoscopies increases the risk of aspiration pneumonia (28, 29), but not bowel perforation or splenic injury (29). Safety is a prerequisite for colorectal cancer screening; endoscopists and anesthesiologists should carefully explain to participants before performing sedated colonoscopy and perform pre-examination assessments to avoid adverse events.

In addition to effectiveness and safety, cost-effectiveness is an important factor to consider when developing a screening strategy. In this study, the full cost coverage group had the best effectiveness, but the highest average colonoscopy cost and highest cost of colonoscopy needed to detect one case of CAN. Additional screening costs may still be economical and preferred in areas where colorectal screening is adequately funded, and a formal cost–benefit analysis is required. However, unsedated colonoscopy is used in many parts of the world (30). The partial cost coverage strategy could be a possible way to increase the participation rate in underfunded areas of CRC screening, with no increase in colonoscopies paid for by the fund since participants pay for sedation themselves. This approach may also be used to help regions that do not already have colorectal cancer screening and quickly establish effective screening strategies at a low financial cost.

This study has several strengths. First, to our knowledge, this is the first study in China to investigate the effect of sedated colonoscopy with different cost coverage on improving compliance with CRC screening in asymptomatic community populations. Second, this study was conducted under the framework of CanSPUC, which used rigorous standards to guarantee the integrity and accuracy of the collected data, including a review mechanism to ensure the quality of data and the development of a data system to monitor all the processes of the study. Third, we evaluated the participation rate, detection rate, and cost of sedation colonoscopy with different cost coverages, and the results were comprehensive.

This study has several limitations. First, for practical reasons, only CRC screening data of the population in Xuzhou were used in this study. Second, due to the limitations of the conditions, the subjects were not randomly grouped in this study, which may have led to selection bias. In addition, only participants who met the high-risk conditions for CRC were recommended to undergo colonoscopy because of examination due to limited resources when CanSPUC was conducted. There may have been a decrease in colonoscopy participation in the average-risk population, but this did not affect the conclusions of this study.

In summary, sedated colonoscopy increased colonoscopy participation rates in both the partial and full cost-covered groups, and the diagnosis rate remained unchanged. The full cost-covered strategy works better but comes with additional costs. A partial cost coverage strategy may be a good way to increase colorectal cancer participation rates and quickly establish a colorectal cancer screening strategy in underfunded areas.

## Data availability statement

The raw data supporting the conclusions of this article will be made available by the authors, without undue reservation.

## Ethics statement

The studies involving human participants were reviewed and approved by the Ethics Committee of Xuzhou Cancer Hospital (approved number: 2018-02-23-H01). The patients/participants provided their written informed consent to participate in this study.

## Author contributions

LiZ, YK, and LaZ conceived and designed the study. YK, YM, HZ, WD, and DD contributed to the acquisition of the data. LiZ, YK, SC, YM, TC, JP, XW, YG, HL, XL, LM, and LaZ were involved in the analysis and interpretation of the data. All authors were involved in the writing, reviewing, and editing of the manuscript. YK, YM, and DD confirm the authenticity of all the raw data. All authors contributed to the article and approved the submitted version.

## References

[1] Sung H Ferlay J Siegel RL Laversanne M Soerjomataram I Jemal A. Global cancer statistics 2020: GLOBOCAN estimates of incidence and mortality worldwide for 36 cancers in 185 countries. CA Cancer J Clin (2021) 71:209–49. doi: 10.3322/caac.21660 33538338

[2] Chen W Zheng R Baade PD Zhang S Zeng H Bray F. Cancer statistics in China, 2015. CA Cancer J Clin (2016) 66:115–32. doi: 10.3322/caac.21338 26808342

[3] Gupta N Kupfer SS Davis AM. Colorectal cancer screening. JAMA (2019) 321:2022–3. doi: 10.1001/jama.2019.4842 PMC7285652 31021387

[4] Brenner H Hoffmeister M Stegmaier C Brenner G Altenhofen L Haug U. Risk of progression of advanced adenomas to colorectal cancer by age and sex: estimates based on 840,149 screening colonoscopies. Gut (2007) 56:1585–9. doi: 10.1136/gut.2007.122739 PMC2095643 17591622

[5] Dekker E Tanis PJ Vleugels JLA Kasi PM Wallace MB. Colorectal cancer. Lancet (2019) 394:1467–80. doi: 10.1016/s0140-6736(19)32319-0 31631858

[6] Lauby-Secretan B Vilahur N Bianchini F Guha N Straif K International Agency for Research on Cancer Handbook Working Group. The IARC perspective on colorectal cancer screening. N Engl J Med (2018) 378:1734–40. doi: 10.1056/nejmsr1714643 PMC6709879 29580179

[7] Chen H Li N Ren J Feng X Lyu Z Wei L. Participation and yield of a population-based colorectal cancer screening programme in China. Gut (2019) 68:1450–7. doi: 10.1136/gutjnl-2018-317124 30377193

[8] Bretthauer M Kaminski MF Løberg M Zauber AG Regula J Kuipers EJ. Population-based colonoscopy screening for colorectal cancer: a randomized clinical trial. JAMA Intern Med (2016) 176:894–902. doi: 10.1001/jamainternmed.2016.0960 27214731 PMC5333856

[9] National Center for Health Statistics. Health, United States, 2019. Hyattsville, MD (2021). Available at: 10.15620/cdc:100685 33818995

[10] Zhang K Yuan Q Zhu S Xu D An Z. Is unsedated colonoscopy gaining ground over sedated colonoscopy? J Natl Med Assoc (2018) 110:143–8. doi: 10.1016/j.jnma.2016.12.003 29580447

[11] Khan F Hur C Lebwohl B Krigel A. Unsedated colonoscopy: impact on quality indicators. Dig Dis Sci (2020) 65:3116–22. doi: 10.1007/s10620-020-06491-0 32696236

[12] Aljebreen AM Almadi MA Leung FW. Sedated vs unsedated colonoscopy: a prospective study. World J Gastroenterol (2014) 20:5113–8. doi: 10.3748/wjg.v20.i17.5113 PMC4009549 24803827

[13] Yan L Liu HZ Lin GZ Liang YR Wang SX Li K. Results of colorectal cancer screening in Guangzhou, 2015. China Caner (2016) 25:422–5. doi: 10.11735/j.issn.1004-0242.2016.06.A004

[14] Zhou Q Li Y Liu HZ Liang YR Lin GZ. Willingness to pay for colorectal cancer screening in Guangzhou. World J Gastroenterol (2018) 24:4708–15. doi: 10.3748/wjg.v24.i41.4708 PMC6224470 30416318

[15] Lew JB St John DJB Xu XM Greuter MJE Caruana M Cenin DR. Long-term evaluation of benefits, harms, and cost-effectiveness of the national bowel cancer screening program in Australia: a modelling study. Lancet Public Health (2017) 2:e331–40. doi: 10.1016/s2468-2667(17)30105-6 29253458

[16] Pornet C Dejardin O Morlais F Bouvier V Launoy G. Socioeconomic determinants for compliance to colorectal cancer screening. a multilevel analysis. J Epidemiol Community Health (2010) 64:318–24. doi: 10.1136/jech.2008.081117 19740776

[17] Zhang J Xu H Zheng L Yu J Chen Q Cao X. Determinants of participation and detection rate of colorectal cancer from a population-based screening program in China. Front Oncol (2020) 10:1173. doi: 10.3389/fonc.2020.01173 32850337 PMC7412959

[18] Montminy EM Karlitz JJ Landreneau SW. Progress of colorectal cancer screening in united states: past achievements and future challenges. Prev Med (2019) 120:78–84. doi: 10.1016/j.ypmed.2018.12.004 30579938

[19] Henley SJ Thomas CC Lewis DR Ward EM Islami F Wu M. Annual report to the nation on the status of cancer, part II: progress toward healthy people 2020 objectives for 4 common cancers. Cancer (2020) 126:2250–66. doi: 10.1002/cncr.32801 PMC7223723 32162329

[20] Kanth P Inadomi JM. Screening and prevention of colorectal cancer. BMJ (2021) 374:n1855. doi: 10.1136/bmj.n1855 34526356

[21] Wender R Brooks D Sharpe K Doroshenk M. The national colorectal cancer roundtable: past performance, current and future goals. Gastrointest Endosc Clin N Am (2020) 30:499–509. doi: 10.1016/j.giec.2020.02.013 32439084

[22] Centers for Disease Control and Prevention. United States cancer statistics colorectal cancer stat bite (2022). Available at: https://www.cdc.gov/cancer/uscs/about/stat-bites/index.htm (Accessed December 19, 2022).

[23] Chen H Li N Shi J Ren J Liu C Zhang Y. Comparative evaluation of novel screening strategies for colorectal cancer screening in China (TARGET-c): a study protocol for a multicentre randomised controlled trial. BMJ Open (2019) 9:e025935. doi: 10.1136/bmjopen-2018-025935 PMC6500225 31005927

[24] Chen H Lu M Liu C Zou S Du L Liao X. Comparative evaluation of participation and diagnostic yield of colonoscopy vs fecal immunochemical test vs risk-adapted screening in colorectal cancer screening: interim analysis of a multicenter randomized controlled trial (TARGET-c). Am J Gastroenterol (2020) 115:1264–74. doi: 10.14309/ajg.0000000000000624 32282342

[25] Chen H Shi J Lu M Li Y Du L Liao X. Comparison of colonoscopy, fecal immunochemical test, and risk-adapted approach in a colorectal cancer screening trial (TARGET-c). Clin Gastroenterol Hepatol (2022) S1542-3565(22):00767–4. doi: 10.1016/j.cgh.2022.08.003 35964896

[26] National Cancer Center, China, Expert Group of the Development of China Guideline for the Screening, Early Detection and Early Treatment of Colorectal Cancer. China Guideline for the screening, early detection and early treatment of colorectal cancer (2020, Beijing). Chin J Oncol (2021) 43:16–38. doi: 10.3760/cma.j.cn112152-20210105-00010 33472315

[27] Liang M Zhang X Xu C Cao J Zhang Z. Anesthesia assistance in colonoscopy: impact on quality indicators. Front Med (Lausanne) (2022) 9:872231. doi: 10.3389/fmed.2022.872231 35911411 PMC9326494

[28] Cooper GS Kou TD Rex DK. Complications following colonoscopy with anesthesia assistance: a population-based analysis. JAMA Intern Med (2013) . 173:551–6. doi: 10.1001/jamainternmed.2013.2908 PMC3987111 23478904

[29] Bielawska B Hookey LC Sutradhar R Whitehead M Xu J Paszat LF. Anesthesia assistance in outpatient colonoscopy and risk of aspiration pneumonia, bowel perforation, and splenic injury. Gastroenterology (2018) 154:77–85.e3. doi: 10.1053/j.gastro.2017.08.043 28865733

[30] Leung FW Aljebreen AM Brocchi E Chang EB Liao WC Mizukami T. Sedation-risk-free colonoscopy for minimizing the burden of colorectal cancer screening. World J Gastrointest Endosc (2010) 2:81–9. doi: 10.4253/wjge.v2.i3.81 PMC2998881 21160707

